# Obesity in Severe COVID-19 Patients Has a Distinct Innate Immune Phenotype

**DOI:** 10.3390/biomedicines11082116

**Published:** 2023-07-27

**Authors:** Ayane de Sá Resende, Yrna Lorena Matos de Oliveira, Mariana Nobre Farias de Franca, Lucas Sousa Magalhães, Cristiane Bani Correa, Kiyoshi Ferreira Fukutani, Michael Wheeler Lipscomb, Tatiana Rodrigues de Moura

**Affiliations:** 1 Health Sciences Graduate Program, Federal University of Sergipe, Aracaju 49060-100, Sergipe, Brazil; yrna.lore@gmail.com (Y.L.M.d.O.); mariananobre51@gmail.com (M.N.F.d.F.); lucas.smagalhaes@hotmail.com (L.S.M.); crisbani@gmail.com (C.B.C.); ferreirafk@gmail.com (K.F.F.); 2 Department of Parasitology and Pathology, ICBS, Federal University of Alagoas, Maceio 57072-900, Alagoas, Brazil; 3 Physiological Sciences Graduate Program, Federal University of Sergipe, São Cristovao 49100-000, Sergipe, Brazil; 4 Department of Pharmacology, University of Minnesota, Minneapolis, MN 55455, USA; lips0046@umn.edu

**Keywords:** obesity, body mass index, COVID-19, SARS-CoV-2, innate immune responses

## Abstract

Obesity alters the capacity of effective immune responses in infections. To further address this phenomenon in the context of COVID-19, this study investigated how the immunophenotype of leukocytes was altered in individuals with obesity in severe COVID-19. This cross-sectional study enrolled 27 ICU COVID-19 patients (67% women, 56.33 ± 19.55 years) that were assigned to obese (BMI ≥ 30 kg/m^2^, n = 9) or non-obese (BMI < 30kg/m^2^, n = 18) groups. Monocytes, NK, and both Low-Density (LD) and High-Density (HD) neutrophils were isolated from peripheral blood samples, and surface receptors’ frequency and expression patterns were analyzed by flow cytometry. Clinical status and biochemical data were additionally evaluated. The frequency of monocytes was negatively correlated with BMI, while NK cells and HD neutrophils were positively associated (*p* < 0.05). Patients with obesity showed a significant reduction of monocytes, and these cells expressed high levels of PD-L1 (*p* < 0.05). A higher frequency of NK cells and increased expression of TREM-1+ on HD neutrophils were detected in obese patients (*p* < 0.05). The expression of receptors related to antigen-presentation, phagocytosis, chemotaxis, inflammation and suppression were strongly correlated with clinical markers only in obese patients (*p* < 0.05). Collectively, these outcomes revealed that obesity differentially affected, and largely depressed, innate immune response in severe COVID-19.

## 1. Introduction

The 2019 coronavirus pandemic (COVID-19), caused by the severe acute respiratory syndrome coronavirus 2 (SARS-CoV-2), had devastating consequences on the mortality and morbidity of afflicted individuals worldwide. Although most patients presented asymptomatic or mild disease states, others developed severe disease, often associated with excessive inflammation and respiratory distress [1,2]. The innate immune system has the crucial role of recognizing the virus and activate inflammatory pathways in order to mount an effective antiviral response and viral clearance [3]. However, evidence has shown the heterogeneity of the immune response in severe COVID-19, which can exhibit hyperinflammatory and/or immunosuppressive phenotypes in effector cells [2,4,5,6,7]. Limited studies have suggested that this altered innate immune response seems to be patient-dependent [2] and the different outcomes of SARS-CoV-2 infection may be influenced by preexisting comorbidities [8,9,10]. Thus, the detection of biomarkers and severity-related immunological alterations are being studied to improve treatments, especially in groups adversely affected by infection with COVID-19, such as patients with obesity.

Obesity is a chronic inflammatory disease often reported to be strongly associated with increased patient admittance into intensive care units (ICU), requiring respiratory support, extended hospitalization time, in-hospital complications, and showing the most frequent comorbidity among severe and fatal cases of COVID-19 [11,12,13,14,15]. SARS-CoV-2 interacts essentially with angiotensin-converting enzyme 2 (ACE2) to invade cells, which is highly expressed in the lungs [16], and also in visceral fat. Thus, adipose tissue in patients with obesity can contribute to the dysregulation of immune cells [9,17,18,19,20]. For instance, Santos e Silva et al. found 17 genes related to innate immune dysfunction in autopsy lungs of COVID-19 patients with obesity, including a higher expression of the suppressive marker programmed death ligand 1 (PD-L1, also known as CD274), antigen-presentation (MHC-class II), cytokine signaling and neutrophil migration, such as CXCR2 (CD182) and antibody-dependent effector receptors, such as the FCGR3A and FCG3B (CD16+). They also found little-to-no presence of neutrophils in more than 80% of the lung tissue sections. These outcomes were not observed in non-obese patients and occurred independent of hypertension or type 2 diabetes [21]. Neutrophils are the primary effector cells among leukocytes to be recruited to infected sites. An elevated neutrophils-to-lymphocytes (N/L) ratio indicates higher absolute neutrophil counts in blood and is associated with the severity of COVID-19 [22,23] and obesity [24,25]. In COVID-19, a heterogeneous population of neutrophils was found, including low-density (LD) and high-density (HD) neutrophils. The LD neutrophils are found among peripheral blood mononuclear cells (PBMC) and can demonstrate pro-inflammatory or suppressive functions in severe COVID-19 [23,26]. HD neutrophils are the mostly widely studied population; however, it is not known whether obesity is associated with these subpopulations in patients with severe COVID-19.

In the absence of any infection, obesity has been reported to be associated with the dysregulation of immunometabolism and skewed functions of monocytes, macrophages, neutrophils, and natural killer (NK) cells [24,25,27,28,29,30]. Complementarily, among several immunological markers, TREM-1, a receptor expressed mainly on the neutrophil’s surface, has also been studied in COVID-19 and other chronic inflammatory diseases as an important target contributing to hyperinflammation [31,32,33]. Moreover, TREM-1 has been previously associated with inflammation in obesity, insulin resistance and other obesity-associated comorbidities [34,35,36].

However, it remains largely elusive as to whether the peripheral innate immune response is distinctly altered in obese patients with severe COVID-19. Despite many efforts, dynamics and alterations in distinct immune markers associated with COVID-19-infected obese patients are not completely elucidated. Thus, these investigations aimed to determine the surface receptors related to activation, inflammation, and suppressive activities of peripheral monocytes, NK cells, and both LD and HD neutrophils in obese and non-obese patients with severe COVID-19 and their association with clinical markers of severity.

Our findings demonstrate a distinct innate immune response, with both suppressive and inflammatory functions related to different leukocyte subpopulations. These phenotypes were strongly associated with obesity in severe COVID-19. The outcomes also highlight innate immune markers that should be explored as targets for treatments of in-hospital obese patients.

## 2. Materials and Methods

### 2.1. Study Design and Participants

Twenty-seven patients were enrolled in the study during treatment in intensive care units (ICU) requiring invasive oxygen support. They were diagnosed with COVID-19-associated pneumonia confirmed by a positive RT-qPCR for SARS-CoV-2 and by a typical chest CT-scan finding. These patients were recruited from two reference public hospitals located in Aracaju (Sergipe), Brazil, between 8 September 2020, and 10 December 2020. Individuals with obesity had not been vaccinated so far. Severity was determined according to the WHO COVID-19 technical guidance (https://apps.who.int/iris/handle/10665/330854 (accessed on 30 August 2020).

One peripheral blood sample was collected from all patients after the ICU admission. All patients were taking corticosteroids (dexamethasone, methylprednisolone and hydrocortisone) as first-line treatment for critically ill patients with COVID-19 due to global recommendations at that time [37,38,39]. We did not have access to dosage, but we were informed that all patients received the same dosage as the hospital’s protocol. Exclusion criteria were as follows: under 18 years old, BMI underweight, having any kind of cancer or auto-immune disease, pregnant women, and subjects that presented issues during flow cytometry experiments. Additionally, patients with a history of chronic corticosteroid therapy prior to COVID-19 treatment were excluded from the study.

Demographic, clinical and laboratory data were retrieved from inpatient health records and are individually presented in Supplementary Table S1. The N/L ratio was calculated from the absolute numbers of neutrophils and lymphocytes. BMI was documented by a nutritionist upon admission. Based on BMI classification by WHO [40], patients were divided into two groups: obese (OB; BMI ≥ 30 kg/m^2^) and non-obese (N-OB; BMI < 30 kg/m^2^).

### 2.2. Flow Cytometry

**Figure 1:**
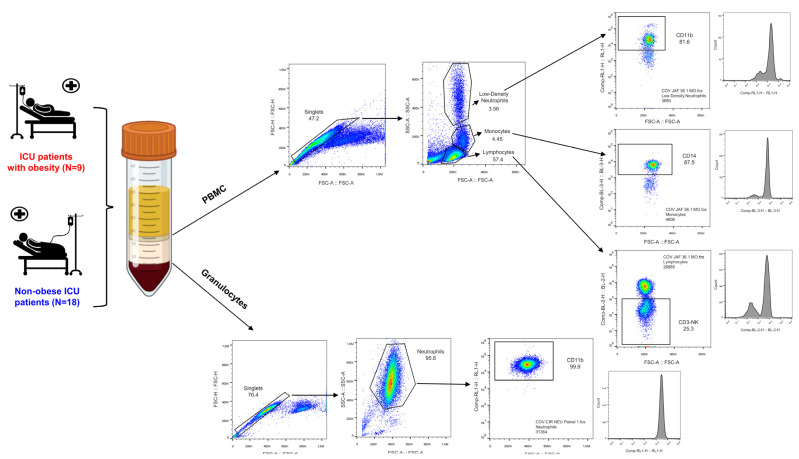
Gating strategies. Firstly, live cells were selected from singlets and, then, each immune cell (monocytes, lymphocytes, LD, and HD neutrophils) was gated according to size (Forward Scatter; FSC-A) and granularity (Side Scatter area; SSC-A) parameters. Separately, CD14+ cells for monocytes, CD3- cells for NK, CD11b+ for LD neutrophils, and CD11b+ and CD11c+ cells for HD neutrophils were selected from different panels, as previously described [41]. Into the gate of CD14+ monocytes, the frequency of positive cells for CD80, CD163, CD274, and HLA-DR and their respective expressions were analyzed. Into the gate of CD3- NK cells, the frequency and expression of CD11c, CD16, and CD56 were analyzed. Into the gate of CD11b+ LD neutrophils, the frequency and expression of CD14, CD274, and HLA-DR were analyzed. Into the gate of CD11b+ HD neutrophils, the frequency and expression of CD16, CD182, and TREM-1 were analyzed. Finally, into the gate of CD11c+ HD neutrophils, the frequency and expression of CD274, CD279, and HLA-DR were analyzed. Confidence intervals were used to report the relative frequency of each immune cell population and fluorescence intensity values are reported as MFI, which were determined by FlowJo according to intensity values of events gated from live cells.

Peripheral blood mononuclear cells (PBMC) and polymorphonuclear cells (PMNC) were isolated from fresh EDTA blood samples by centrifugation (400× *g*, 25 °C, 35 min) with Ficoll-Paque PLUS™ (Sigma-Aldrich, San Luis, MO, USA). After washing, 1 × 10^6^ of these cells were stained with the following monoclonal antibodies: CD14 PerCP 5.5 and CD274 PE (BD Biosciences, San Jose, CA, USA) and CD80 FITC, CD163 PE-Cy7, and HLA-DR Alexa 700 (ThermoFisher Scientific, Waltham, MA, USA) for Monocytes; CD3 PE, CD11c PerCP 5.5, CD16 FITC and CD56 PE-Cy7 (BD Biosciences, San Jose, CA, USA) for Natural Killer (NK) cells; CD14 PerCP 5.5 and CD274 PE PE (BD Biosciences, San Jose, CA, USA), and HLA-DR Alexa 700 (ThermoFisher Scientific, Waltham, MA, USA)) for Low-Density (LD) Neutrophils; and CD11b APC and CD16 FITC (BD Biosciences, San Jose, CA, USA), CD182 PE-Cy 5.5 (ThermoFisher Scientific, Waltham, MA, USA)), TREM-1 PE (BioLegend, San Diego, CA, USA), CD11c PerCP 5.5, CD274 PE and CD279 FITC (BD Biosciences, San Jose, CA, USA) and HLA-DR Alexa 700 (ThermoFisher Scientific, Waltham, MA, USA)) for High-Density (HD) Neutrophils. The description of the antibodies used in flow cytometric experiments is detailed in Supplementary Table S2. Samples were acquired in Attune™ NxT Flow Cytometer software (version 2.4, ThermoFisher Scientific, Waltham, MA, USA). Data were analyzed using FlowJo software (version V10, Treestar Inc., Ashland, OR, USA). Figure 1 illustrates gating strategies. Statistic values were used to report the relative frequency of immune cell populations and fluorescence intensity values are reported as MFI.

Thirteen healthy adults with a negative RT-qPCR were included as an internal control for flow cytometric experiments. Of these, 46.15% were women, with a mean age of 33.85 ± 7.16 years old and a BMI of 24.73 ± 3.24 kg/m^2^.

### 2.3. Inflammatory Measurement

Sera were obtained from peripheral blood after centrifugation (1600× *g*, 25 °C, 10 min) and stored at −80 °C until analysis. The levels of IL-6 (ThermoFisher Scientific, Waltham, MA, USA) were measured using enzyme-linked immunosorbent assays kits according to the manufacturers’ instructions, and plates were read in a spectrophotometer Epoch BioTek (Agilent, Santa Clara, CA, USA).

### 2.4. Statistical Analysis

Whether distributions are normal was evaluated by the Shapiro–Wilk test and data are presented as mean and standard deviation or median and 25–75th interquartile range. Simple linear regression was applied to evaluate whether BMI is correlated with the levels of peripheral immune cells from blood leukocytes. Student’s *t*-test with Welch’s correction or Mann–Whitney test was applied to clinical data in order to investigate differences between OB and N-OB groups.

For immune receptors and phenotypes, multiple Mann–Whitney tests were applied to compare OB and N-OB groups, followed by the Benjamini, Krieger, and Yekutieli False Discovery Rate correction. At the time of data collection, we were struggling in the first wave of SARS-CoV-2 infection, and patients with obesity were mainly women and older than non-obese patients [42]. Thus, for comparisons that showed a significant difference, data distribution was log-transformed and a linear regression was applied with age and sex as cofactors. Moreover, within the OB group, younger (<65 years old) and older (>65 years old) patients were compared using Student’s T-test with Welch’s correction or Mann–Whitney test to explore whether there are significant differences related to age. Significant differences were visualized with volcano plots and heatmaps.

To analyze whether the expression of receptors and phenotypes was distinctly associated with clinical markers, mostly white and red blood cells and IL-6 levels, Spearman’s rank correlation coefficient was applied. Strong correlations were considered when Spearman’s r coefficient was ≤−0.70 or ≥0.70.

*p*-values less than 0.05 were considered significant. Statistical analyses and figures were conducted in GraphPad Prism software (version 9.4,San Diego, CA, USA) and R Studio version 4.1.2.

## 3. Results

### 3.1. Participants’ Characteristics

**Figure 2:**
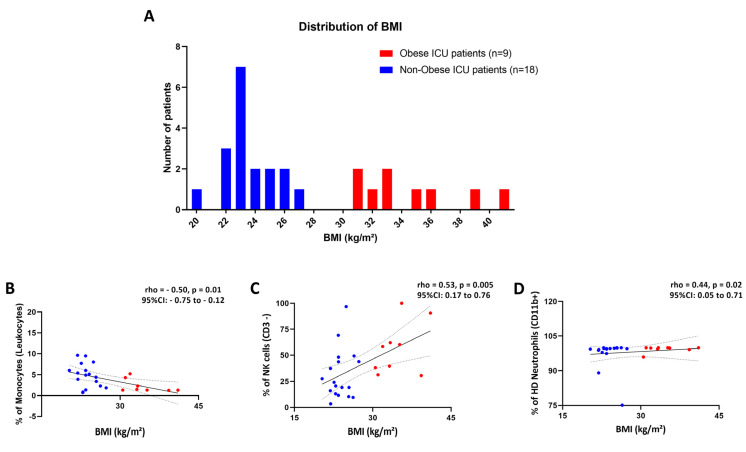
Body mass index (BMI) is associated with peripheral innate immune cells’ frequency. Peripheral blood was collected from 27 SARS-CoV-2 severely infected patients (obese = 9 and non-obese = 18). (**A**) Histogram of BMI distribution among COVID-19 patients. (**B**) Spearman’s R correlation between BMI and the relative frequency (%) of peripheral monocytes, (**C**) NK cells and (**D**) High-density (HD) neutrophils. Red points are representing patients with obesity and blue points are non-obese patients. Legend: %: relative frequency; 95%IC: 95% confidence interval; BMI: body mass index.

All COVID-19 patients had been treated in the ICU and the clinical data shown in Table 1 highlights the state of both groups. BMI and age were significantly different between groups (*p* < 0.01; Table 1). One patient with obesity had a BMI of class III (>40 kg/m^2^; Figure 2A). Five non-obese patients were overweight as classified by BMI scale (25–29.9 kg/m^2^; Figure 2A). Five patients with obesity (55.5%) and four non-obese patients (22.2%) were over 65 years of age. One patient with obesity and one non-obese patient were habitual smokers. In addition, OB patients had a higher body temperature on the day of blood collection compared to N-OB patients (*p* = 0.03, Table 1).

### 3.2. Innate Immune Phenotype Differentiated Patients with Obesity from Non-Obese Patients with Severe COVID-19

Firstly, levels of peripheral monocytes were significantly lower in Obese (OB) compared to the Non-Obese (N-OB) group (*p* = 0.03; Table 1). The frequency of peripheral monocytes was negatively correlated with BMI, as observed in Figure 2A, while peripheral levels of NK and HD neutrophils were positively correlated (Figure 2B–D). The frequency of LD neutrophils did not correlate with BMI (*p* = 0.31).

**Figure 3:**
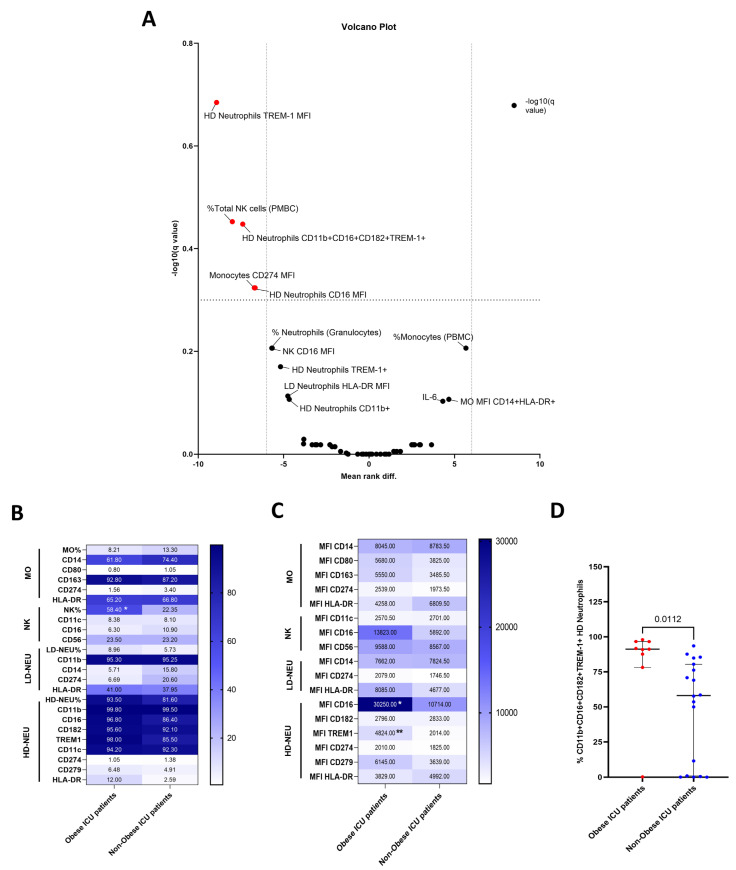
Severe COVID-19 patients with obesity showed increased levels of cell surface receptors for the innate immune response. (**A**) A volcano plot of 58 nonredundant parameters highlights immune markers (red points) that differentiate obese from non-obese severe patients (*p* < 0.05; multiple tests by Mann-Whitney with FDR correction). Heatmap demonstrates (**B**) higher frequency of both circulating Total NK cells and (**C**) higher expression (by mean fluorescence intensity; MFI) of CD274 on CD14+ monocytes and CD16 and TREM-1 on CD11b+ HD neutrophils in severe patients with obesity. (**D**) The inflammatory phenotype (CD11b+CD16+CD182+TREM-1+) on HD neutrophils was significantly higher in patients with obesity compared to non-obese. Panels (**B**,**C**) show the medians and Panel (**D**) also shows the interquartile (25–75th). Comparisons were performed by the Mann–Whitney test. * *p* < 0.05, ** *p* < 0.01 between obese and non-obese patients. Legend: %: relative frequency; HD: high-density neutrophils; ICU: intensive care units; LD: low-density neutrophils; MFI: mean fluorescence intensity.

Then, the next step was analyzing the flow cytometric data and the volcano plot shown in Figure 3A highlights the immune markers that statistically distinguished the OB and N-OB groups. In detail, the OB group revealed a significantly higher frequency of circulating NK cells (*p* = 0.01) and increased frequency of the inflammatory phenotype CD11b+CD16+CD182+TREM-1+ on HD neutrophils (*p* = 0.01) compared to the N-OB group (Figure 3B and Figure 3D, respectively). Moreover, the expression of the CD274 on CD14+ monocytes (*p* = 0.04), and both the CD16 (*p* = 0.03) and TREM-1 (*p* = 0.005) on CD11b+ HD neutrophils were higher among OB compared to N-OB patients (Figure 3C). When these significant outcomes were adjusted for sex and age, only the frequency of NK cells (p-adjusted = 0.04), the MFI of CD274 on monocytes (p-adjusted = 0.03) and the MFI of TREM-1+ on HD neutrophils (p-adjusted = 0.03) remained significantly increased in OB patients with severe COVID-19.

To evaluate possible age influence in the cell’s phenotype, studies also compared young (<65 years old) and older (>65 years old) patients within the OB group and no significant differences were found in any immunological or clinical variable included in this study (*p* > 0.05; Supplementary Table S5).

### 3.3. Patients with Obesity Showed a Substantial Number of Strong Correlations between Their Immunophenotype and Clinical Markers

**Figure 4:**
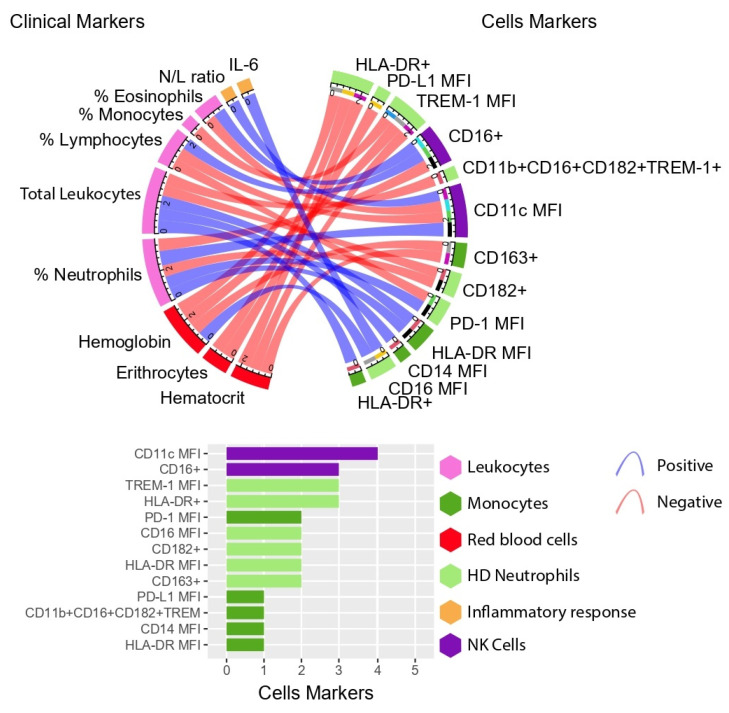
Several innate immune markers were uniquely associated with the clinical status of severe COVID-19 patients with obesity (n = 8). Only significant (*p* < 0.05) and strong correlations (rho = <−0.70 or >0.70) between clinical variables (right side of the circles) and immune markers (left side of the circles) were included. The legend colors indicate the parameters to which the clinical and cellular markers belong. This figure was created in R studio with the Circus script. Right down the circles, there is a ranking of the correlations count of each immune marker. Legend: %: relative frequency; N/L ratio: neutrophils to lymphocytes ratio; HD: high-density neutrophils; MFI: mean fluorescence intensity; PD-L1: programmed death-ligand 1; PD-1: Programmed death-1 receptor; IL-6: interleukin 6.

To gain sensitivity to a possible obesity-associated immunophenotype in critically ill COVID-19 patients, we then investigated whether the surface receptors were associated with clinical data in each COVID-19 group. Uniquely, patients from the OB group showed several strong correlations between the surface receptors of monocytes, NK cells and HD neutrophils and routine laboratory data, such as white blood cells, red blood cells and IL-6 (Figure 4).

For instance, CD14+ monocytes expressing CD163 showed a positive association with hemoglobin (rho = 0.72, *p* = 0.03) and hematocrit (rho = 0.73, *p* = 0.02) levels. Moreover, the expression of the CD14+ (rho = −0.77, *p* = 0.01) on monocytes, as well as the frequency (rho = −0.83, *p* = 0.005) and expression (rho = −0.82, *p* = 0.007) of HLA-DR on CD14+ monocytes, were negatively associated with total leukocytes. The MFI of HLA-DR on CD14+ monocytes were also negatively correlated with the frequency of blood neutrophils (rho = −0.73, *p* = 0.02) (Figure 4).

Moreover, NK cells’ receptors showed strong correlations with several white blood cells, as shown in Figure 4. NK cells expressing CD11c on their surface negatively correlated with neutrophils (rho = −0.93, *p* = 0.0002), while positively correlating with both eosinophils (rho = 0.73, *p* = 0.03) and lymphocytes (rho = 0.75, *p* = 0.02). The clinical marker of severity, neutrophil-to-lymphocyte (N/L) ratio, also showed a negative correlation with this surface receptor (rho = −0.72, *p* = 0.03). However, the frequency of CD16+ on NK cells positively correlated with neutrophils (rho = 0.80, *p* = 0.01) and with N/L ratio (rho = 0.67; *p* = 0.04), while negatively correlating with eosinophils (rho = −0.78, *p* = 0.01) and lymphocytes (rho = −0.73, *p* = 0.02).

HD neutrophils’ surface receptors were highly associated with clinical markers, as shown in Figure 4. For example, IL-6 levels of patients with obesity were negatively associated with CD16 expression on CD11b+ HD neutrophils (rho = −0.80, *p* = 0.01). Moreover, the MFI of CD182 showed a positive association with both total leukocytes (rho = 0.73, *p* = 0.03) and blood neutrophils (rho = 0.73, *p* = 0.03). Positive associations were also detected between the MFI of TREM-1 with blood monocytes (rho = 0.74, *p* = 0.02), hemoglobin (rho = 0.80, *p* = 0.01) and hematocrit (rho = 0.73, *p* = 0.02) levels. Furthermore, the frequency of HD neutrophils expressing the inflammatory phenotype CD11b+CD16+CD182+TREM-1+ is positively associated with total leukocytes (rho = 0.75, *p* = 0.02). In addition, the frequency of HLA-DR+ on HD neutrophils was positively correlated with red blood cells, such as erythrocytes (rho = 0.78, *p* = 0.01), hemoglobin (rho = 0.80, *p* = 0.01) and hematocrit (rho = 0.82, *p* = 0.007). Finally, CD274 expression on HD neutrophils was positively associated with erythrocytes (rho = 0.81, *p* = 0.008), while the expression of CD279 on these cells was negatively associated with circulating neutrophils (rho = −0.80, *p* = 0.01) and positively associated with blood lymphocytes (rho = 0.72, *p* = 0.03). Results also revealed a moderate association between the expression of CD279 on HD neutrophils and the N/L ratio (rho = −0.67, *p* = 0.04).

Notably, the N-OB group showed fewer correlations between immune and clinical markers in comparison with the OB group (OB: 27 correlations vs. N-OB: 17 correlations, Supplementary Tables S6 and S7) and no strong correlation was detected. For instance, only the frequency of CD80 on CD14+ monocytes was negatively associated with erythrocytes (rho = −0.54, *p* = 0.03). Similar to the OB group, NK cells expressing CD16+ positively correlated with blood neutrophils (rho = 0.55, *p* = 0.03) and the expression of HLA-DR on CD11b+ HD neutrophils was positively correlated with hemoglobin (rho = 0.57, *p* = 0.02) and hematocrit (rho = 0.57, *p* = 0.02). The frequency of CD279+ on these cells showed a positive association with erythrocytes (rho = 0.52, *p* = 0.04) and CD16+ on HD neutrophils a positive association with total leukocytes (rho = 0.64, *p* = 0.007). On the other hand, data revealed the MFI of CD182 was positively associated with lymphocytes (rho = 0.59, *p* = 0.01) and negatively associated with the N/L ratio (rho = −0.53, *p* = 0.03). Blood lymphocytes were also positively associated with the MFI of TREM-1+ on HD neutrophils (rho = 0.52, *p* = 0.04).

## 4. Discussion

In agreement with Santos e Silva et al. [21], our findings reveal immune markers associated with obesity. Specifically, BMI was associated with peripheral innate immune frequency in severe COVID-19 patients and, importantly, with the immune response towards suppressive and inflammatory activities related to different leukocyte subpopulations. In addition, several innate immune receptors involved in antigen-presentation (HLA-DR), phagocytosis and antibody-dependent response (CD16), effector functions (CD11c), chemotaxis (CD182), suppressive activity (PD-L1 and PD-1), inflammation (TREM-1) and hemoglobin clearance (CD163) were strongly associated with clinical markers of severity in patients with obesity.

Kooistra and colleagues evaluated a similar population of OB and N-OB COVID-19 patients. The authors demonstrated that there was no significant difference between groups by analyzing laboratory and inflammation markers (IL-6, IL-8, TNF-α, IFN-γ, IP-10 and IL-10), detecting only difference in relation to body temperature [43]. Regardless of these differences, both the volunteers from Kooistra’s study and the patients in this present study had higher body temperatures. The fever mechanism can be mediated by cytokines through the IL-1/IL-6/PGE2 axis, as well as by the recognition of pathogen-associated molecular patterns (PAMPs) in the surface of innate immune cells [44]. In the present study, inflammatory activity by neutrophils was also detected, characterized by the increased expression of TREM-1, which acts in conjunction with Toll-like receptor 4 [36], suggesting that these cells may contribute to the higher temperature observed in OB patients. Besides, in this present study, a significant association was found between immune cells and BMI, especially when these cells were determined by their surface markers (CD3- for NK, and CD11b+ for neutrophils). Thus, our study reinforces the importance of investigating the immunophenotype in this population with obesity.

Alterations of peripheral innate immune function were documented in obese individuals without an infection, mainly, characterized by non-classical monocytes [45], NK cells with impaired cytotoxic and imbalanced expression of activating and inhibiting receptors [46,47], and increased N/L ratio, with a predominance of inflammatory activity by neutrophils compared to non-obese individuals [24,47]. Accordingly, this present study revealed a significant reduction of circulating monocytes, which were expressing high levels of suppressive PD-L1, as well as a higher frequency of peripheral NK cells. Moreover, HD neutrophil phenotype indicated an increased inflammatory activity. In agreement, Grewal and Buechler demonstrated that adipokines, mainly leptin, resistin and galectin-3, are closely involved in peripheral monocytes and neutrophil responses, which contributes to complications during the course of COVID-19 in patients with obesity. The authors also highlight that the overall inflammation in severe cases, as observed by routine markers, masks obesity-related inflammatory processes, which hampers treatment efficacy [10]. Therefore, despite comparable disease severity between COVID-19 groups, these observations indicate that patients with obesity have a distinct innate immunophenotype in severe COVID-19 that should be explored as biomarkers.

In accordance with our study, Zulu et al. [8] have observed an abnormal innate immune response among COVID-19 patients with obesity, mainly a negative correlation between BMI and frequency of peripheral monocytes. Previous studies that broadly investigated immunopathogenesis in SARS-CoV-2 infection demonstrated that monocytes suffer dynamic changes according to severity [5,48,49,50] and a decrease of circulating monocytes is implicated in less effective and highly suppressive functions in the periphery [48,49,51], such as an impaired response to viral stimulation [51]. Besides, the upregulation of the PD-L1/PD-1 axis has been reported to be dysfunctional in chronic infections, such as HIV and hepatitis B and C viruses [52], as well as in severe cases of COVID-19 [7]. Additional data from our laboratory demonstrated a significant reduction of total CD3+ lymphocytes (*p* = 0.02) and a higher frequency of T naïve helper expressing PD-1 (CD3+CD4+CD28+CD279+; *p* = 0.04) in this population of COVID-19 patients with obesity compared to N-OB group (unpublished data). Thus, we speculated that there is a crosstalk in peripheral immune response between monocytes and T helper cells, which is supported by previous studies [53,54].

In this context, Giamarellos-Bourboulis et al. have found that severe patients with respiratory failure can display opposite phenotypes, with either macrophage activation syndrome, which contributes to the known cytokine storm, or very low HLA-DR expression with loss of functions [5]. However, these authors did not investigate the influence of comorbidities on the immunophenotype. In our study, the expression of this antigen-presentation (HLA-DR) receptor was not significantly different between severe COVID-19 groups (*p* = 0.07), although we found a negative association between this surface receptor on CD14+ monocytes and both total leukocytes and circulating neutrophils exclusively in patients with obesity (Figure 4). Our outcomes highlight that obesity is associated with severity. Nonetheless, whether peripheral monocytes have negative implications on the immune response in severe COVID-19 patients with obesity needs further investigation, although obesity is suggested to have an impaired response to therapies, such as vaccines [55,56].

Interestingly, a significantly higher peripheral frequency of total NK cells was observed in patients with obesity and it was also reported in severe obesity without infections [46]. In COVID-19, studies demonstrated the opposite, with a significant, or at least sustained, reduction in NK cell frequency associated with COVID-19 severity [5,50,57]. In our population with obesity, NK cells expressing CD11c+ and the frequency of CD16+ NK cells are contrarily associated with the N/L ratio, a clinical marker of severity. An increase in the N/L ratio indicates a higher inflammatory response, which corroborates with the cytotoxic phenotype of CD16+ NK cells. Severity influences and may reduce the effector functions of NK cells, which may explain the heterogeneous findings observed in the scientific literature [5,50,57,58]. Additionally, our study underlines the obesity influence on the innate immune response. The N/L ratio is also implicated in infectious diseases [23], and in obesity-associated features without an infection condition [24,25].

Neutrophil activity may also coordinate inflammatory signaling in OB patients with severe COVID-19 as observed by the significantly higher expression of TREM-1 on HD neutrophils, in congruence with the hyperinflammatory phenotype (CD11b+CD16+CD182+TREM-1+) that was strongly and positively associated with total leukocytes in this population. Moreover, the negative relationship between the programmed death-1 receptor (PD-1 or CD279) and the N/L ratio supports the predominance of inflammatory activity by HD neutrophils. A previous study including patients with obesity has shown HD neutrophils with enhanced phagocytosis, respiratory oxidative burst, degranulation, and neutrophil extracellular (NET) formation in severe COVID-19 patients [23]. Neutrophils are often reported to be higher in severe COVID-19, contributing to tissue damage with NET release [22,33]; however, outcomes from other studies, including Santos e Silva’s work, suggest a dysfunctional response in this population [21,23,24].

In addition, TREM-1 has been consistently investigated in inflammatory and infectious diseases, since its main function is to amplify inflammatory responses [31,32,59]. TREM-1 expression and the expression of haptoglobin-hemoglobin scavenger receptor (CD163) on monocytes and HLA-DR on HD neutrophils were also positively associated with red blood parameters in the OB group, which may indicate activation related to the endothelial damage. Indeed, there is a higher risk for endothelial damage and related complications in this population [60], and innate immune cells are suggested to be involved in coagulation and phagocytosis of red blood cells in COVID-19 [23,61]. DAMPs are also more frequently observed in obese individuals and, thus, can influence neutrophils’ phenotype and COVID-19 severity [62,63]. These overall features were different from the N-OB group, e.g., CD182 negatively correlated with N/L ratio and TREM-1 related to blood lymphocytes, which strengthens the different immune responses between groups regardless of comparable disease severity.

Patients were also under corticosteroid treatment at blood collection and there was a non-statistical, although interesting, difference in duration of the treatment with corticosteroids between groups. This may be associated with the observation that patients with obesity had a trend to lower IL-6 levels (*p* = 0.06, Table 1). In this context, Pinski et al. have shown, through transcriptional and proteomic analysis, upregulated genes from peripheral blood associated with inflammatory activity by neutrophils in severe COVID-19 patients with obesity and diabetes, both young and aged individuals under corticosteroid treatment [64], which is in accordance with previous reports [21,48,53,56]. Corticosteroids are widely used routinely in ICU to suppress inflammation in mechanically-ventilated patients [37,38,39]. Previous studies have already reported different responses according to the severity and to chronic diseases, such as obesity [21,48,53,56]. In accordance, it was already documented that obesity can impair the immune response to corticosteroids [65,66,67,68,69], as well as vaccine efficacy [52,53,70,71,72]. In this context, the increase in TREM-1 expression compared to non-obese patients suggests an inflammatory activity by neutrophils, while patients with obesity were under mean longer treatment with corticosteroids (Table 1). Whether there is a threshold of illness severity at which corticosteroids are indicated, or a time- or dose-dependent response in this population, are the questions that remained in the literature. Thus, our results underpin the need for more studies targeting immune response in this population.

Finally, some limitations are acknowledged as we did not perform a longitudinal analysis and we did not obtain information on survival or death from all patients, which limits our understanding of whether immune cells from patients with obesity are responsive to treatment or dysfunctional. In addition, there were more deaths among patients with obesity from whom we obtained information (Supplementary Table S1). Patients were also under corticosteroid treatment at blood collection and there was a non-statistical, although interesting, difference in duration of the treatment between groups (Table 1). Although we have performed a statistical correction for age and gender, we also highlight the differences in age, gender and metabolic diseases (i.e., DM type 2) as limitation. In addition, our sample size is quite small and devoid of statistical power. Our findings and our observational design should be carefully interpreted as they also reflect peripheral innate immune responses. Besides, the inclusion of a group of obese patients non-infected with SARS-CoV-2 and a healthy group could expand the understanding of obesity’s influence on the immune response. Furthermore, BMI does not provide an indication of fat distribution or differentiation between muscle and adipose tissue [73]. Although BMI has been widely used as an indirect indicator of obesity and showed an association with the severity of COVID-19 [8,11,15], visceral fat has a major influence on systemic inflammation compared to subcutaneous fat [73]. Thus, the inclusion of waist circumference and other complementary parameters to BMI could enhance the understanding of how obesity modulates COVID-19. As this field of immunity is little explored in patients with obesity, further studies considering the markers evaluated here are needed. Our outcomes support the importance of deeply investigating the relationship between obesity and immunology, since there is an important association of obesity with mortality in COVID-19, even in younger patients [11].

## 5. Conclusions

In summary, BMI is associated with the frequency of monocytes, NK cells and neutrophils in peripheral blood. Specifically, the frequency of peripheral NK cells, the expression of PD-L1 on CD14+ monocytes, and TREM-1 on CD11b+ HD neutrophils were the major discriminators between OB and N-OB patients with severe COVID-19, regardless of age and sex. Our outcomes revealed that obesity impacts immune response, even in the absence of clear differences in clinical status. We also highlighted several surface receptors related to effector responses strongly and exclusively associated with clinical markers in patients with obesity. These phenotypes can help in monitoring in-hospital patients with obesity, since flow cytometry has become useful in clinical practice, for therapeutic decisions and vaccine development [10,53,55,57].

## Figures and Tables

**Table 1:** Characteristics and clinical data of ICU COVID-19 patients.

Variables	Obese (BMI ≥ 30; n = 9)	Non-Obese (BMI < 30; n = 18)	Normal Range
Characteristics			
Women, n (%)	7.00 (77.78)	9.00 (42.85)	-
Age, years	67.67 ± 11.08 *	50.67 ± 20.62	-
BMI, kg/m^2^	34.56 ± 3.63 *	23.64 ± 1.85	18.50–24.90
Medical history			
Hypertension, n (%)	3.00 (33.33)	6.00 (33.33)	-
CVD, n (%)	1.00 (11.11)	1.00 (5.55)	-
Type 2 DM, n (%)	4.00 (44.44)	3.00 (16.66)	-
COPD, n (%)	1.00 (11.11)	1.00 (5.55)	-
CLD, n (%)	0.00 (0.00)	1.00 (5.55)	-
Laboratory data			
Erythrocytes, ×10^6^/uL	3.50 [3.17–3.66]	3.19 [2.83–3.51]	3.90–5.20
Hemoglobin, g/dL	9.23 ± 1.06	8.88 ± 1.17	11.70–15.70
Hematocrit, %	28.71 ± 4.97	26.24 ± 4.12	36.00–47.00
Platelets, x10^3^/uL	184.30 ± 117.20	218.60 ± 141.00	150.00–450.00
Total Leukocytes, ×10^3^/uL	13.79 [12.21–18.18]	13.10 [9.65–25.13]	4.00–11.00
Neutrophils, %	85.30 ± 9.75	83.23 ± 10.17	40.00–70.00
Neutrophils count, ×10^3^/uL	11.80 [9.60–16.18]	11.23 [7.45–22.99]	1.60–8.00
Eosinophils, %	1.80 [0.20–5.06]	0.72 [0.18–2.97]	1.00–5.00
Basophils, %	0.50 [0.40–0.67]	0.40 [0.28–0.66]	0.00–1.00
Lymphocytes, %	3.94 [2.67–9.57]	6.64 [2.05–14.40]	20.00–40.00
Lymphocytes count, ×10^3^/uL	0.55 [0.51–1.63]	1.20 [0.32–1.77]	0.90–4.00
Monocytes, %	1.40 [1.30–3.80] *	4.90 [2.08–6.86]	2.00–12.00
N/L Ratio ^&^	23.39 [5.08–34.49]	12.23 [5.34–45.51]	-
Body temperature, celsius	37.80 [37.40–38.00] *	37.00 [37.00–37.20]	36.00–36.90
PaO_2_/FiO_2_^&^	150.80 [119.40–193.40]	178.90 [155.10–220.00]	450.00–500.00
ALT, U/L	30.25 ± 19.65	41.17 ± 18.27	7.00–55.00
AST, U/L	30.25 ± 15.44	40.00 ± 17.12	8.00–48.00
Serum Urea, mg/dL	94.00 [57.00–99.50]	79.50 [38.50–120.30]	10.00–45.00
Serum Creatinine, mg/dL	0.71 [0.57–0.93]	1.11 [0.65–1.95]	0.60–1.20
Serum Sodium, mEq/L	146.80 [132.10–149.00]	136.10 [135.80–142.00]	135.00–145.00
Serum Potassium, mEq/L	4.10 [3.57–5.08]	4.61 [3.58–4.95]	3.50–5.50
Na/K Ratio ^&^	34.09 [26.12–41.73]	30.93 [26.85–38.87]	-
Total Bilirubin, mg/dL	0.45 ± 0.29	0.41 ± 0.24	0.30–1.00
Pro-inflammatory markers			
CRP, mg/dL	99.80 [66.78–130.80]	108.50 [82.75–159.50]	<0.80
IL-6, pg/mL	33.35 ± 10.32	52.98 ± 35.99	1.50–7.00
Clinical outcome			
Days under corticosteroid treatment	15.00 [2.00–33.00]	7.00 [4.25–22.50]	-
In-hospital death, n (%)	7.00 (77.78)	7.00 (38.89)	-

Data are presented as mean and standard deviation or median and 25–75th interquartile range according to normality distribution. Legend: ICU: intensive care unit; DM: type 2 diabetes mellitus; COPD: chronic obstructive pulmonary disease; CLD: chronic liver disease; CVD: cardiovascular disease; N/L ratio: neutrophils-to-lymphocytes ratio; ALT: alanine aminotransferase; AST: aspartate aminotransferase; CRP: C-reactive Protein; Na/K: sodium-to-potassium ratio; IL-6: interleukin 6. The percentage of white blood cells was calculated according to the total leukocytes of each patient. ^&^ arbitrary units. * *p* < 0.05 by Student’s *t*-test with Welch’s correction or Mann-Whitney between Obese and Non-obese groups.

## Data Availability

Clinical data of each participant is available in Table S1. Flow cytometric data can be requested by sending an e-mail to the corresponding author.

## Supplementary Materials

The following supporting information can be downloaded at: https://www.mdpi.com/article/10.3390/biomedicines11082116/s1, Table S1: Demographic, clinical and laboratory characteristics of the study groups; Table S2: Description of the antibodies used in flow cytometry experiments; Table S3: Spearman R’s correlation with Body Mass Index; Table S4: Relative numbers of PBMC cells and Polymorphonuclear Neutrophils and its respective Mean Fluorescence Intensity (MFI) of severe COVID-19 patients; Table S5: Relative numbers of PBMC cells and Polymorphonuclear Neutrophils and its respective Mean Fluorescence Intensity (MFI) between younger and older than 65 years old; Table S6: Matrix of correlations between immunological surface receptors and clinical markers within Obese patients; Table S7: Matrix of correlations between immunological surface receptors and clinical markers within Non-obese patients.
